# Polarized Catalytic Polymer Nanofibers

**DOI:** 10.3390/ma12182859

**Published:** 2019-09-05

**Authors:** Dinesh Lolla, Ahmed Abutaleb, Marjan A. Kashfipour, George G. Chase

**Affiliations:** 1 Biosciences and Water Filtration Division, Parker-Hannifin Corporation, Oxnard, CA 93030, USA; 2 Department of Chemical Engineering, Jazan University, Jazan 45142, Saudi Arabia; 3 Department of Chemical and Biomolecular Engineering, The University of Akron, Akron, OH 44325, USA

**Keywords:** electrospinning, polarization, heterogenous catalysis, PVDF, phenol, cyclohexanone

## Abstract

Molecular scale modifications were achieved by spontaneous polarization which is favored in enhancements of β-crystallization phase inside polyvinylidene fluoride (PVDF) nanofibers (NFs). These improvements were much more effective in nano and submicron fibers compared to fibers with relatively larger diameters. Metallic nanoparticles (NPs) supported by nanofibrous membranes opened new vistas in filtration, catalysis, and serving as most reliable resources in numerous other industrial applications. In this research, hydrogenation of phenol was studied as a model to test the effectiveness of polarized PVDF nanofiber support embedded with agglomerated palladium (Pd) metallic nanoparticle diameters ranging from 5–50 nm supported on polymeric PVDF NFs with ~200 nm in cross-sectional diameters. Scanning Electron Microscopy (SEM), Transmission Electron Microscopy (TEM), Atomic Force Microscopy (AFM), Energy Dispersive X-Ray Spectroscopy (EDX), Fourier Transform Infrared Spectroscopy (FTIR) and other analytical analysis revealed both molecular and surface morphological changes associated with polarization treatment. The results showed that the fibers mats heated to their curie temperature (150 °C) increased the catalytic activity and decreased the selectivity by yielding substantial amounts of undesired product (cyclohexanol) alongside with the desired product (cyclohexanone). Over 95% phenol conversion with excellent cyclohexanone selectivity was obtained less than nine hours of reaction using the polarized PVDF nanofibers as catalytic support structures.

## 1. Introduction 

Metal and metal oxide (M/MO) nanoparticles (NPs) have excellent catalytic properties [1,2,3,4,5]. However, M/MO NPs cause high-pressure drop in fixed-bed systems. They also possess poor mechanical strength and chemical stability. In addition, M/MO NPs are not a perfect choice for flow reactors since they are so difficult to separate from the fluids. M/MO NPs tend to aggregate resulting in the reduction of their surface area to volume ratio and thus causing lower catalytic performance. Furthermore, the selectivity of M/MO NPs is usually low because these NPs exhibit a lack of specificity for the desired reactions in complex reactions [6,7,8,9].

To enhance the catalytic properties of the M/MO NPs and to overcome the previous mentioned limitations, M/MO NPs have been supported on natural and synthetic polymers. Polymers with specific functional groups is one way to improve the catalytic activity and selectivity of the M/MO NPs [6,7,8,9]. Catalysts made of M/MO NPs supported on polymers have been investigated in different reactions, including oxidation, cyclic polymerization, Suzuki cross-coupling reaction, and hydrogenation. Table 1 shows some examples of metal nanoparticles supported on polymer catalysts. 

Catalysts fabricated from M/MO NPs supported on polymers (M/MO NPs-Polymer) combine the excellent properties of both NPs and polymers. Different factors such as the type of functional groups and surface properties of the polymeric support, size and concentration of the NPs, and the fabrication technique control the final catalytic properties of the M/MO NPs-Polymer [9]. Polymeric functional group is the main factor that affects the binding capacity of metal ions/complexes/precursors to fabricate the M/MO NPs-Polymer catalyst. NPs nature and size are influenced by the fabrication technique and the morphological properties of the polymer like the surface area and the porosity [9].

M/MO NPs can be dispersed on different polymeric forms such as spherical beads, granules, membranes, fibers, and others. Nanofibers (NFs) are defined as fibers with a diameter equal to or less than 100 nm. This definition is usually relaxed in the industry to include all fibers that have submicron diameters [17]. NFs possess many excellent properties such as small fiber diameter, high aspect ratio (length to diameter ratio), huge surface area to volume ratio or mass ratio, high and controllable porosity, small pore size and great mechanical stiffness, and tensile strength along the fibers which make them excellent materials for different engineering applications [18,19,20,21,22,23].

Surface area of catalysts is a very crucial feature in determining the performance of heterogeneous catalysts. Hence, nanofibrous materials with very high porosity and surface area are encouraging catalytic support materials. Excellent catalytic activity and high capacity for the attachment or release of different chemical species [17] are provided by the NFs due to their high surface area. Nanofibrous materials also show low resistance to the flow of liquids and gases and feasibility of adapting to various geometries [24]. 

Functionalized polymers are a unique class of organic materials. They are robust, chemically stable and willing to chemical modification depending on the intended application need. For functionalized polymeric fibers, projection of the electrical fields from the fibers into the pore openings is necessary for the electrostatic attraction mechanism to be effective. The stronger the electric field the farther the effect extends into the pore spaces and the more effective is the mechanism for particle capture [25,26]. 

In this research, the functionalized polyvinylidene fluoride (PVDF) fibers were polarized to enhance the adsorption of the reactants on the surface of the polymeric nanofibrous support. PVDF were chosen to be the catalytic support since the dipole moments are distributed along the fiber axis and the electrostatic attractive forces integrate along the length of the fiber segments [27].

Hydrogenation of phenol to cyclohexanone was used as a model for this catalyst. The reaction is very important in the industry to produce cyclohexanone, which is a raw material for nylon industries [28,29]. It is very difficult to obtain excellent cyclohexanone selectivity at high phenol conversion since the desired product (cyclohexanone) is very active and can be easily hydrogenated to undesired product (cyclohexanol) as shown in Equation (1). One way to obtain great cyclohexanone selectivity at high phenol conversion is by using an efficient catalyst.

Phenol + 2H_2_ → Cyclohexanone + H_2_ → Cyclohexanol (1)

M/Mo NPs supported on electrospun polymers have been proven as practical heterogeneous catalysts. For example, Soukup et al. [30] have prepared palladium and platinum NPs supported on poly (2,6-dimethyl-1,4-phenylene) oxide electrospun membranes by electrospinning and wet impregnation. To the best of our knowledge, no one has modified the electrospun catalytic support surface by polarization. Here, palladium (Pd) NPs supported on polarized polymeric PVDF NFs, were fabricated using very cost-effective techniques. The catalytic performance of the prepared polarized catalysts were then tested in the phenol hydrogenation reaction and compared to non-polarized catalysts.

## 2. Catalytic Support Preparation

### 2.1. Electrospinning of PVDF Fibers

#### 2.1.1. Materials Used 

PVDF, a semi-crystalline fluoropolymer is well known by its trade name Kynar. Kynar-761 grade with an average molecular weight of approximately 550,000, density 1.78 g/cm^3^, melting point ranging between 165–172 °C, and melt viscosity 23–29 (as per MSDS) was generously supplied by Archema Inc (King of Prussia, PA, USA). Acetone, N, N-Dimethylformamide (DMF) were purchased from Sigma Aldrich (St. Louis, MO, USA) and were used without any further purification. Palladium (Pd) black (520810) was also purchased from Sigma Aldrich.

#### 2.1.2. Preparation of Electrospun Fibers

Electrospinning is among one of the most predominantly reported techniques from past two decades to derive slender two-dimensional, multilayered structured non-woven fiber mats from many synthetic polymers. It gained research and commercial interest due to its simplicity, ease of maintenance, and low cost of production of small quantities. Electrospinning can produce continuous long single fibers with relatively small diameters ranging from a few nanometers to about 10 microns. Electrospinning parameters can be varied to alter the properties of the fibers and fiber mats (fiber diameters, internal porosity, surface charges, and formation of beads) that may affect filter performance. 

A total of 10 wt.% PVDF was dissolved in a blend of (1:1 volume ratio) acetone and DMF. DMF was used to reduce the solvent volatility which is favored in formation of submicron fibers. PVDF is immiscible in DMF: Acetone mixture at room temperature, but it can form a homogenous solution when heated to 70 °C. The polymer solutions were heat stirred for 20 minutes before electrospinning until it was clear and transparent. 

**Figure 1:**
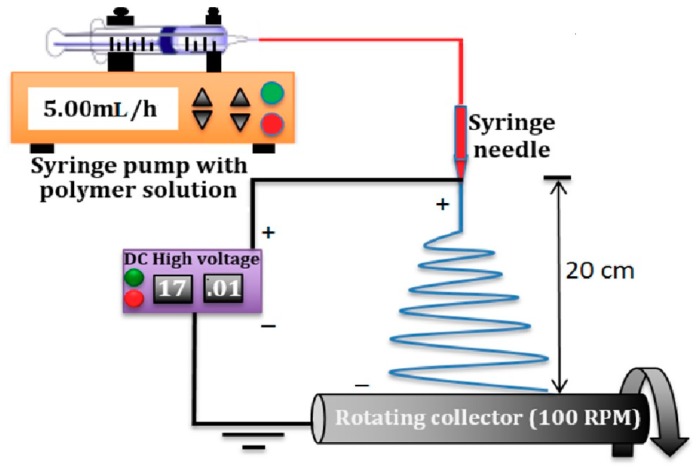
Schematic of syringe pump electrospinning station with rotating cylindrical collector.

Figure 1 is the schematic of the electrospinning setup utilized in this research. A 5 mL BD plastic syringe was preheated to 70 °C, filled with polymer solution. A syringe was connected by Teflon™ tubing to a blunt stainless steel 21-gauge needle using a male and female end connector. The polymer solution was pumped using an automated syringe pump (SP220i World Precision Instruments, Sarsota, FL, USA). The syringe pump helps by delivering the polymer solution with a specific preset flowrate of 5 mL/h. Electrospinning conditions are shown in Table 2. 

A high voltage DC power supply (ES60P, Gamma High Voltage Research, Ormond Beach, FL, USA) was used to generate the voltage potential listed in Table 2 between the syringe needle and the grounded non-stick aluminum foil on the rotating drum. The needle was incrementally moved from left to right by 5 cm to different positions along the axis of the rotating collector, with 5 mL solution deposited at each position, until the drum surface was covered with a mat of fibers with an average specific weight of 20 g/m^2^. All the electrospun sheets were heated for 2 h at 70 °C in an oven to evaporate residual solvents.

#### 2.1.3. Design of Polarization Holder

**Figure 2:**
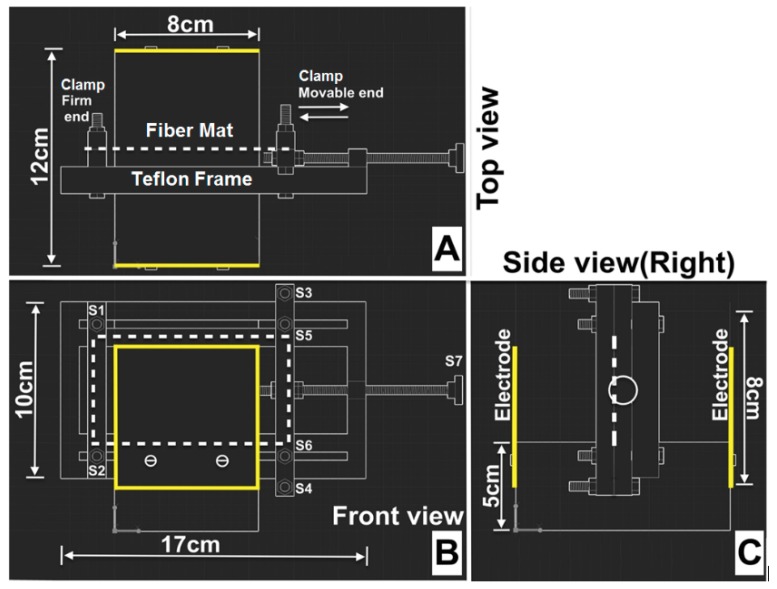
Perspective views of custom-made polarization device designed using Autodesk®-AutoCAD®-2015. Orientation of the electrospun fiber mat is indicated by the dotted white lines and aluminum electrodes are indicated by solid yellow lines. Copyright reprints from [18].

Detailed polarization steps and associated surface charge of the PVDF fibers are reported in our previous publication [18]. Figure 2 is a custom-made device, used to perform polarization experiments.

### 2.2. Reaction Methodology

#### 2.2.1. Batch Reactor Design

**Figure 3:**
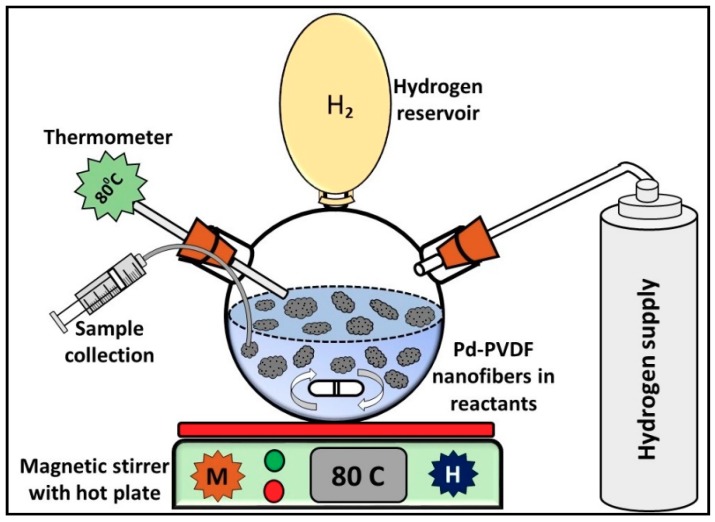
Schematic of batch reactor, not drawn to scale.

A custom-made batch reactor was utilized to study the performance of the prepared catalysts. The batch reactor was built from a 3-neck 250 mL round bottom flask. The three openings of the flask were connected to a hydrogen (H_2_) cylinder, a balloon, syringe for sample collection, and a thermometer as shown in Figure 3. The H_2_ cylinder was connected to the bottom flask via 1/8 stainless steel tubing. The balloon was used to store H_2_ in the reactor. The thermometer was immersed inside the reactor to measure the actual temperature of the reactants. Mixing and heating of reactants and catalyst was obtained by using a magnetic stirrer (Thermo Fisher Scientific, Waltham, MA USA).

Before the start of any reaction, the system was flushed with H_2_ at least three times to remove air from the system. After flushing the system, the balloon was filled with H_2_ and the reaction started. A product sample was taken at specific times using a syringe. It is suggested to make sure that there are no leaks in the system. A contact thermometer was used to ensure the reactant temperature was constantly maintained at 80 °C.

#### 2.2.2. Reaction Samples Analysis 

The reaction products were analyzed using a gas chromatography (GC, SHIMADZU GC-17A, Columbia, MD, USA), equipped with Ionized Flame Detector (IFD) and HP-FFAP column^12^. The GC was connected with an autosampler to obtain faster and more accurate analysis. GC was operated under the following specification: Injection temperature (220 °C), injection pressure (8 kPa), total flow rate (58 mL/min) with (1.12 mL/min) in the column, carrier gas (nitrogen and air), column temperature (120 °C), and detector temperature (240 °C).

The analysis of phenol (Sigma Aldrich, St. Louis, MO, USA), cyclohexanone and cyclohexanol were performed in two steps. First, a calibration curve for each compound was constructed through the following steps: A) Preparation of five different known concentrations of the compound, B) dilution of the prepared samples with ethanol (1:1 volume) to obtain better peaks resolutions, C) analysis of each diluted sample in the GC to obtain the peak area for each concentration, D) plotting peak areas versus concentrations for the compound.

Second, after running the reaction, a product sample was taken at three different times, 3, 6, and 9 hours. The sample was then analyzed in the GC and the GC gives the peak area of each compound, phenol, cyclohexanone, and cyclohexanol, and the peak area was converted into concentration using the constructed calibration curves. After calculating the concentrations from the calibration curves, Equations (2) and (3) were used to calculate the phenol conversion (X_Phenol_) and the cyclohexanone selectivity (S_Cyclohexanone_).

(2) $$\mathrm{Phenol}\;\mathrm{conversion}\;X_{\mathrm{Phenol}}=\frac{C_{\mathrm{Phenol}\;\mathrm{Inirial}}-C_{\mathrm{Phenol}\;\mathrm{Final}}}{C_{\mathrm{Phenol}\;\mathrm{Initial}}}$$

(3) $$\mathrm{Cyclohexanone}\;\mathrm{selectivity}\;S_{\mathrm{Cyclohexanone}}=\frac{C_{\mathrm{Cyclohexanone}}}{C_{\mathrm{Cyclohexanone}}{+C}_{\mathrm{Cyclohexanol}}}$$

where $C_{\mathrm{Phenol}\;\mathrm{Initial}}$: Concentration of phenol (mol/L) before the beginning of the reaction.

$C_{\mathrm{Phenol}\;\mathrm{Final}}:$ Concentration of phenol (mol/L) in the samples that were taken at different reaction times. 

$C_{\mathrm{Cyclohexanone}}$: Concentration of cyclohexanone (mol/L) in the samples that were taken at different reaction times. 

$C_{\mathrm{Cyclohexanol}}$: Concentration of cyclohexanone (mol/L) in the samples that were taken at different reaction times.

### 2.3. Preparation of Metal Nanoparticles Supported on Polymeric Nanofibers

**Figure 4:**
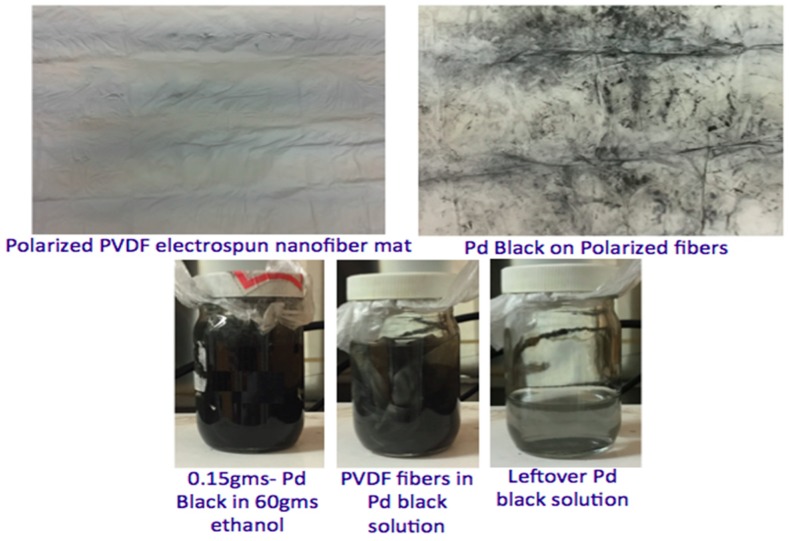
Preparation of electrospun polarized palladium (Pd)/PVDF.

The catalytic particles were dispersed on the electrospun fibers using the traditional wet impregnation technique. A total of 0.15 g of Pd catalytic NPs was dissolved in 60 g of ethanol for one hour using a magnetic stirrer at room temperature to obtain uniform dispersion of particles. Later, PVFD fibers were immersed and stirred for five hours in the Pd–ethanol solution to impregnate Pd particles on fibers. Figure 4 shows the impregnation of Pd on PVDF NFs.

## 3. Results and Discussions

### 3.1. Morphological Analysis

Field Emission Scanning Electron Microscopy (FESEM, JSM-7401F JEOL Ltd., Peabody, MA, USA) imaging and corresponding energy-dispersive X-ray (EDX) analysis were conducted using a Scanning Electron Microscope-TESCAN-LYRA-3 model XMU FIB-FESEM equipped with a AMETEX Energy Dispersive X-ray Analyzer. All the electron microscopy images were recorded at an accelerating voltage of 5 kV. A low specimen current of 2.0 nA was used. EDX analysis was used to identify Pd catalytic particles composition dispersed on the PVDF fibers, X-ray spot probe was placed on top of the location of interest for 30 s. Analysis of this EDX data provided comparisons of Pd distributions at different locations of the fibers.

**Figure 5:**
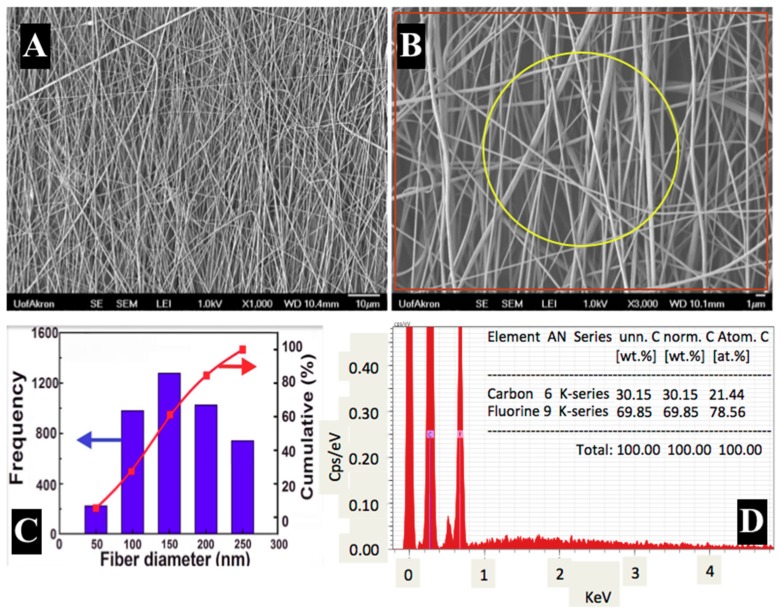
SEM electron micrographs, associated fiber size distributions and EDX elemental compositions of PVDF electrospun fibers. Figure 5A, B are low and high magnification images of PVDF nanofibers, Figure 5C is fiber size distribution and 5D is elemental composition of the electrospun PVDF nanofibers.

Figure 5A represents a tiny sample of as-spun PVDF fibers in low magnification SEM image and Figure 5B is a high magnification image of the same sample used for elemental composition analysis. From the SEM images, the morphologies of the fibers were aligned, smooth, and continuous without any beads. Many open pore structures along the mat were also seen throughout the specimens and these micro-voids favored by entrapping the Pd-particles in between layers of fibers. Figure 5D is the EDX spectra and elemental composition of the electrospun fibers highlighted in a yellow circle in Figure 5B. The data indicates the fibers in a given sample consist of 30.15 wt.% of Carbon and 69.85 wt.% of Fluorine. Hydrogen atom composition is not detected by EDX due to the lack of core electrons. 

The Fibraquant 1.3 fiber analysis software (nanoScaffold Technologies LLC, version 1.3, Chapel Hill, NC, USA) was used to interpret the SEM images to determine the size distributions as shown in Figure 5C. Nine SEM images were analyzed from different areas of the fiber mat to obtain 5000 data points which determined average fiber size distribution from several hundreds of fibers and the results were reported in terms of frequency and % cumulative against given fiber diameters. In Figure 5C, fiber diameters were observed in the range of 50–290 nm with an average diameter of 196 ± 54 nm. 

### 3.2. FT-IR Analysis

Ever since the discovery of piezoelectric property exhibited by polyvinylidene fluoride (PVDF), polymer and material scientists showed utmost interest on the processing of the semi-crystalline polymer properties and five distinct polymorphs well known as, α (TGTG’), β (TTTT), γ (T_3_GT_3_G’), δ, and ε. Fourier transform Infrared Spectroscopy (FTIR) techniques are widely used to characterize structural properties of PVDF.

Gregorio et al. [30] and Lopes et al. [31] proposed a quantification technique to calculate the relative amounts of electroactive β-phase and γ-phase by considering a three-phase system as two-phase system by neglecting α-phase. These systems are considered as a complex and unreliable technique for exact phase quantification. Xiaomei et al. [32] developed an integrated phase quantification methodology for mixed peak systems like PVDF by sorting out more than 100 prior publications from different research groups across the world.

**Figure 6:**
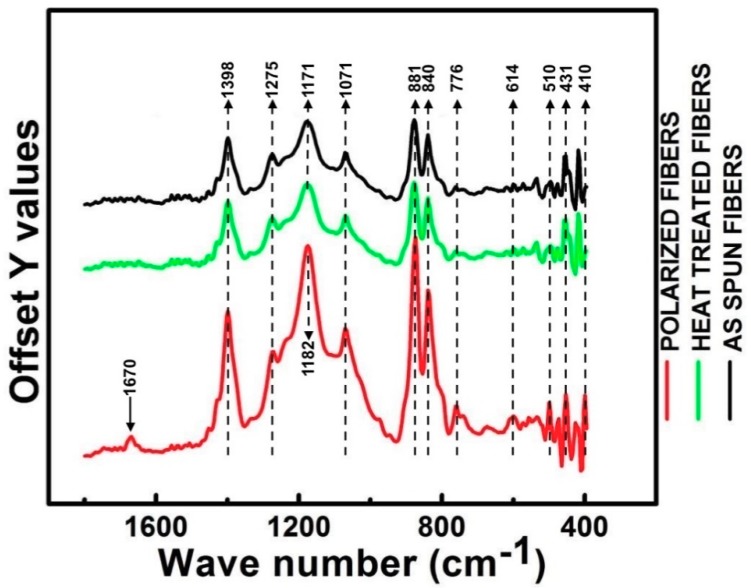
A comparison of FTIR spectra profiles in the fingerprint region between 400–1800 cm^−1^.

Twelve distinct absorption peaks were observed in the range of 400–1800 cm^−1^ wavelength as shown in Figure 6. Only α, β, and γ polymorphs were observed in all the three samples. These exclusive absorption peaks were used to characterize the crystalline structures of different PVDF samples used in this research [18]. From Figure 6, peaks at 410, 614, and 1071 belong to α-phase, 510, 840, 881, 1171–1182, 1275, and 1670 cm^−1^ belong to β-phase, and 431, 1398–1404 cm^−1^ are pertaining to γ-phase. There is a controversy among the absorption peak displayed at 776 cm^−1^ weather if it belongs to β or γ phase, but the majority of the researchers voted for β-phase [32].

Due to mechanical stretching and the strong electric field applied during the electrospinning process, electrospun PVDF fibers most likely produce fibers with higher electroactive β-phase which is quite evident from Figure 6. No significant changes were observed in molecular orientation due to thermal treatment. However, polarized fibers showed a surprisingly sharp increase at absorption peaks located at 776, 840, 881, and 1071 cm^−1^. Strong absorption signal is a direct evidence of polarization induced molecular orientation changes in PVDF fibers. A new absorption peak at 1670 cm^−1^ (β-phase) was observed in polarized fibers, 1670 was not found in both as-spun and heat-treated fibers. Lolla et al. [23] studied this phenomenon by molecular simulation PVDF NFs with fewer molecules in a typical cross-section and attributed this phenomenon for macroscopic molecular orientations.

Only one peak at 1171 cm^−1^ in both as-spun and heat-treated fiber showed a slight shift to 1182 cm^-1^ in polarized fibers. Peak shifting to higher wave numbers indicates a decrease in mass of the molecule as the vibrational frequency is inversely proportional to the mass of the vibrating molecule. Typically, same or similar atomic bonds in different molecules will typically absorb within the same, specific frequency ranges usually do not show any alteration in phase. Among all the 12 peaks, 1275 cm^−1^ (β-phase) was considered as the weakest peak. This peak was seen in all the three samples and Xiaomei et al. observed this peak in raw PVDF powder as well.

### 3.3. Catalytic Characterization

**Figure 7:**
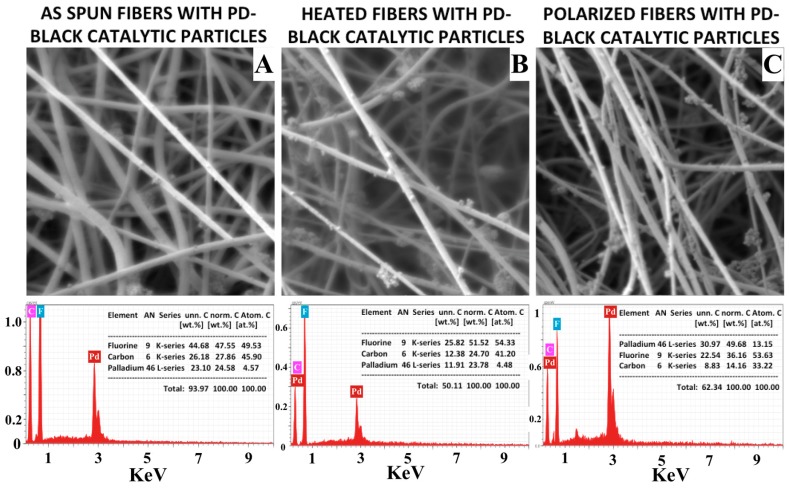
SEM micrographs of catalytic particles embedded on to (**A**) as-spun, (**B**) heat-treated, and (**C**) polarized PVDF fibers and corresponding EDX spectra analysis of elemental composition on the bottom.

EDX data shows as-spun fibers and heat-treated fibers have 4.57 and 4.48% atomic composition of Pd particles, respectively. On the other hand, EDX analysis report on polarized fibers shows 13.15% Pd composition but the fact is that all the three samples have the same wt.% of catalytic particles on the whole sample with irrespective of the compositions reported in Figure 7. Variation in concentration difference from EDX elemental compositions are not a direct representation of the quantity of catalyst introduced during the reaction. Particle composition from the SEM and EDX composition analysis tend to vary significantly especially when particle dispersion is not uniform, and the analyzed area is too localized to derive firm conclusions.

**Figure 8:**
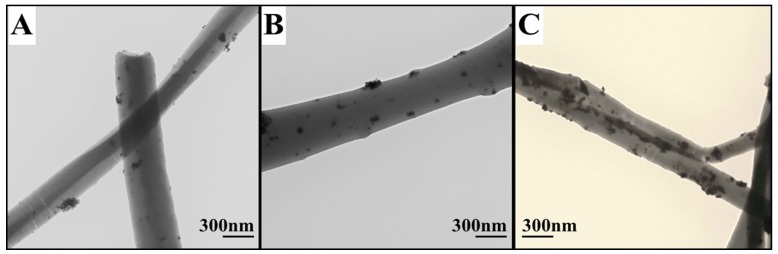
TEM micrographs of catalytic particles embedded on to (**A**) as-spun, (**B**) heat-treated, and (**C**) polarized PVDF fibers.

The dispersion of the Pd catalytic particles in PVDF fibers was examined using TEM analysis. Electrospun fibers were dispersed in ethanol using a sonicator and a tiny drop of suspension of fibers in ethanol was dripped onto a copper grid supported with carbon lacey film. Sonication was performed at 30 kHz for five minutes, lower sonication frequencies were chosen to mitigate particle migration from fiber structure. Sonication frequencies (>80 kHz) for prolonged exposure time resulted in substantial particle detachment and change in crystal geometry. Grid was dried in an oven at 70 °C to evaporate ethanol. This process leaves a substantial amount of fibers on grids for TEM analysis. Figure 8 clearly shows Pd particles (black spots on fibers) distributed all over the fiber. Conclusively, the particles were dispersed in a non-uniform pattern and many particles were agglomerated in to lumps of about 50–70 nm. This particle accumulation on fiber surfaces were seen highly in polarized fibers as shown in Figure 8C.

**Figure 9:**
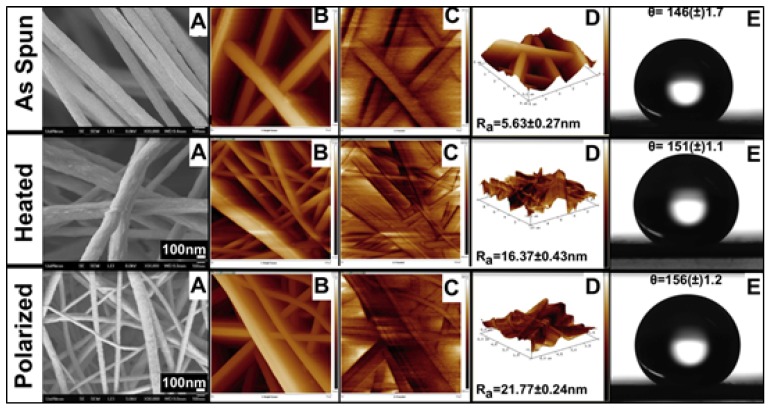
SEM (**A**), AFM (**B**,**C**,**D**), and DSA (**E**) characterizations of electrospun PVDF fibers.

High magnification SEM images reported in Figure 9A show surface textural information. It is clearly visible from the SEM images that surface roughness is greatly increased on the fibers with respect to thermal and electrical treatments. However, it is technically not possible to quantify the increase in roughness on the fibers by SEM imaging due to additional layer of sputtering on the surface of the filers.

AFM analysis was employed to determine actual increase in surface roughness and electric potentials of the fibers. Figure 9B–D shows surface topography, electric potentials, and 3D-overlay of both topography and potential mapping of three samples. Average surface roughness R_a_ reported in Figure 9D was calculated from ten different data points from different samples. The PVDF fiber mats were characterized for their wettability using water contact angles in air. Water droplet on electrospun, heat-treated, and polarized PVDF fibers are shown in Figure 9E. All the three fiber samples showed contact angles greater than 145°.

It was observed that as the surface roughness, polarity of the fibers increased the wettability of the samples as reported in Figure 9E. At least five independent readings were averaged to determine the average contact angle of the fiber mats. As-spun PVDF fibers displayed hydrophobic nature with an average contact angle of θ = 146 ± 1.7 increased to θ = 156 ± 1.2 (super hydrophobic) in polarized fibers. Increase in hydrophobicity favored by allowing more phenol adsorption during the catalytic reaction by simultaneously repelling the water molecules.

### 3.4. Phenol Conversion Reactions

**Figure 10:**
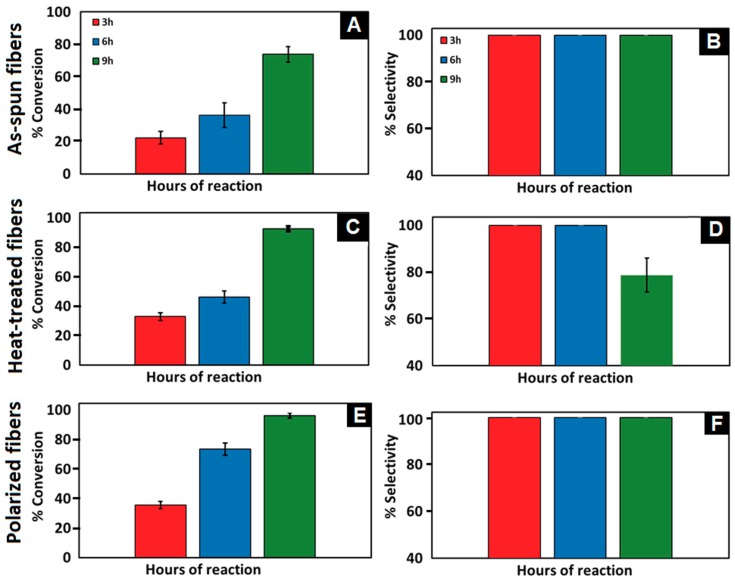
Conversion of phenol (**A**,**C**,**E**) and selectivity of cyclohexanone (**B**,**D**,**F**) using as-spun, heat-treated, and polarized Pd/PVDF electrospun fibers.

The effect of polarization on the catalytic performance was studied by testing of Pd NPs supported on PVDF NFs (5 wt.% of Pd with respect to phenol). The same amount of the polymer and the metal particles were used in as-spun, heat-treated, and polarized fiber preparation. The reaction conditions were 75 mL of 20 wt.% aqueous phenol, 1 atm pressure of H_2_, and 80 °C temperature. Three 5 mL samples were collected using a syringe every three hours. The results reported in Figure 10 are the summary of the conversions of phenol and selectivity of cyclohexanone using as-spun, heat-treated, and polarized Pd/PVDF catalyst from three individual experiments.

As-spun Pd/PVDF yielded very low catalytic activity of 73 ± 4.76% of overall conversion after nine hours of the reaction. Thus, since the catalytic activity was low (<80%), the as-spun catalyst is not an effective catalyst even though it showed excellent selectivity. Heat-treated fibers exhibited similar characteristics as as-spun fibers for the first six hours. Heat-treated fibers recorded a conversion ratio of 32 ± 2.58 and 46 ± 4.16% with excellent selectivity for three and six hours, respectively. Using heat-treated fibers yielded high catalytic activity of 100% overall conversion after nine hours of the reaction. Formation of undesired byproducts (cyclohexanol) occurred after nine hours of reaction time using heat-treated Pd/PVDF resulted in reduction of overall selectivity of the reaction. Selectivity of the product is notably reduced to 78 ± 7.26% due to the formation of cyclohexanol.

Phenol conversion using the polarized catalyst was 73 ± 4.86% in the first six hours of the reaction with excellent cyclohexanone selectivity as shown in Figure 10F, and this is almost equal to nine hours conversion of as-spun catalyst. Figure 10E showed that polarized fibers are the most active supporting structures by yielding the highest product conversion among all tested metals. Phenol conversion of 96.3 ± 1.68% was achieved after nine hours of the reaction. Moreover, the polarized fibers showed excellent selectivity since no undesired products were detected using the described GC method. It is believed that the polarized PVDF helps to affectively adsorb phenol giving high conversion towards the desired reaction and to desorb the cyclohexanone product from the catalytic surface as soon as it is formed which helps to deactivate the undesired reaction resulting in high cyclohexanone selectivity.

It is worth to mention that the liquid product turned to black when using as-spun and heat-treated catalysts due to leaching of Pd catalytic particles to the final product. However, no color change was observed during the whole reaction time when using the polarized catalyst. Leaching of the catalyst into the final product requires additional separation processes, which significantly increases the cost of operation. 

## 4. Conclusion

Nanofibrous materials have many outstanding characteristics that make them excellent candidates for different engineering applications such as filtration and catalysis. Electrospinning is a very efficient technology to fabricate nanofibers (NFs). In this study, a homemade electrospinning setup was utilized to fabricate polyvinylidene fluoride (PVDF) NFs. The PVDF NFs was polarized to enhance their catalytic properties. Three different PVDF samples (as-spun, preheated, and polarized PVDF) were tested to study the effect of polarization on the catalytic performance. Hydrogenation of phenol to cyclohexanone was chosen as a reaction model in the study due to its industrial importance and complications. Preliminary results showed that polarization of the PVDF nanofibrous exhibited enhanced catalytic activity and selectivity. Over 95% conversion with excellent cyclohexanone selectivity was achieved after nine hours of reaction when using polarized Pd/PVDF nanofibers. It is very essential to conduct more research to quantify particle migration, binding strength, effect of change in catalyst, solvent composition, and reusability.

## Figures and Tables

**Table 1:** Examples of catalysts fabricated from metals supported on polymer.

Polymer Support	Metal	Reaction	Reference
Polyaniline (PANI)	Palladium	Suzuki coupling	[10]
Polypyrrole/Polyacrylonitrile (PPy/PAN)	Palladium	Hydrogen production from ammonia borane	[11]
Polyvinylidene fluoride-co-hexafluoropropylene (PVDF-HFP)	Palladium	Hydrogenation of Phenol	[12]
Polyaniline	Palladium	Suzuki reaction	[13]
Poly(N-vinylimidazole)	Ruthenium	Olefin metathesis reactions	[14]
Polyamides	Palladium	Hydrogenation of alkadienes and alkynes	[15]
Polyamides	[RhCI(CO)_2_]_2_, PdC1_2_(PhCN)_2_, PtCI_2_(PhCN)_2_, and RuC1_2_(bipy)_2_	Hydrosilylation of isoprene and 2-methyl-l,3-pentadiene	[16]

**Table 2:** Electrospinning conditions of polyvinylidene fluoride (PVDF) fibers.

Polymer Solution Concentration (wt/wt%)	Solvent Ratio (Acetone: DMF)	Applied Voltage (kV)	Tip to Collector Distance(cm)	Drum Rotation speed (RPM)	Syringe Flow Rate (mL/h)
10%	50:50	17	20	100	5
